# Conversations between women with vulval lichen sclerosus: a thematic analysis of online forums

**DOI:** 10.1186/s12905-021-01223-6

**Published:** 2021-02-17

**Authors:** Gemma L. Bentham, Kristyn Manley, Shehrazad Halawa, Lucy Biddle

**Affiliations:** 1 Royal United Hospitals, Bath, UK; 2 University Hospitals Bristol Trust, Bristol, UK; 3 Derriford Hospital, Plymouth, UK; 4 Bristol University Medical School, Bristol, UK

**Keywords:** Vulval dermatoses, Lichen sclerosus, Women’s health, Patient experience, Health inequalities, Thematic analysis, Online research, Online forums, Sexual health, Social isolation, Peer support, Self-management, Patient-centred care

## Abstract

**Background:**

Vulval lichen sclerosus (VLS) is a common condition. Despite this, there is a paucity of research investigating the impact on women’s lives. Some women with VLS utilise online forums to discuss their priorities and concerns. This dialogue gives insight into the experiences of women living with VLS.

**Methods:**

We identified the most popular public forums containing discussions between women with VLS. Inductive, thematic analysis was applied to 202 online posts spanning a six-year period.

**Results:**

Five key themes were identified. Theme 1 pertained to difficulties with diagnosis. Women experience frequent delays and misdiagnosis. They report health care professionals (HCPs) with poor knowledge of their condition and some that were dismissive of their symptoms. Upon diagnosis women expressed relief and frustration. Theme 2 related to rationalisation and validation of their experience. Women expressed a desire to know why they were affected, what caused their symptoms and gain reassurance. Theme 3 dealt with women’s motivation to control their condition. Women want to know what triggers a flare-up so they can limit their relapses. They want to self-manage their condition and have an active role in partnership with HCPs. Theme 4 related to women sharing and seeking advice from the forums. The lived experiences of other women is valued by fellow sufferers. In particular, women are keen to try other treatments, conventional and alternative. The final theme related to the social repercussions of the condition. Sociocultural factors may prevent women from talking about their condition to friends, family and HCPs. They feel embarrassed by their symptoms. Some women reported relationship breakdown as a repercussion of the disease.

**Conclusions:**

Improving the knowledge of HCPs with regards to VLS may reduce problems with diagnosis. In addition, delivering improved women’s health education in schools may reduce the taboo attached to women’s health. This may empower women to talk about their condition and seek help sooner. Once diagnosed, clinicians with the appropriate expertise should care for women with VLS. Women should be encouraged to take an active role in managing their condition in partnership with clinicians. Future research priorities include identifying the aetiology, triggers for flare-ups and novel therapies.

## Introduction

Lichen sclerosus (LS) is a chronic, inflammatory condition affecting the skin. In women, it has a predilection for the vulva. Vulval LS is common with an estimated prevalence of 1.7–3% [1, 2]. However, the true prevalence is probably higher due to under-recognition and under-reporting [3]. The condition causes itching, pain and scarring which can lead to difficulties with intercourse, urination and defaecation. As a result, women commonly experience a deterioration in their quality of life [4]. Women can suffer for many years without recognition of their condition as a consequence of delayed diagnosis [5]. Upon diagnosis, therapy is targeted at symptom control using high-potency steroids [2] with no prospect of cure. There is a scarcity of research investigating vulval LS [6]. Consequently, the aetiology is poorly understood, there are limited treatment options and little is known about the effect of the condition upon women’s lives. Moving forward, the recently published results of the Lichen Sclerosus Priority Setting Partnership (LSPSP) [7] will raise the profile of LS. The LSPSP aims to direct researchers towards the questions of the highest priority as determined by those affected by LS.

This study aimed to identify what women with LS choose to discuss with each other. This can lend insight into the topics women regards as significant, including living with their condition, the impact upon their lives and issues relating to diagnosis and management of their condition. Such insights are particularly valuable since, given the intimate nature of LS, many women are inhibited from engaging in open discussion about their experiences with healthcare providers. Appreciating the perspective of women living with LS will enable healthcare professionals to deliver patient-centred care that addresses the concerns of women living with this condition.

## Methods

The use of online forums enables researchers to gain access to a large volume of data. This data is organically occurring and allows the observation of real interactions in a non-obtrusive way, akin to some ethnographic research [8]. This is the ideal data source for identifying the patient perspective without imposing any constraints upon the conversation. Online forums also allow for personal anonymity, leading to some security when discussing sensitive or intimate issues. Furthermore, without the perception of being observed, women are at liberty to discuss the topics they consider important. The use of online forums as a dataset, enabled the research team to satisfy the study aim: What are the issues that women with LS choose to discuss with each other?

The forums used in this study were selected by performing an Internet search using the search term “Lichen Sclerosus Discussion”. The Google search engine was selected and the search was performed in January 2019 when the market share of Google in the UK was 92% [9]. In accordance with ethical guidance from the British Psychological Society, we selected the first two largest independent UK websites that did not require membership to view the forums discussions, so called “open forums” [10]. These websites targeted women and encouraged discussions on a wide range of topics. The websites had a high volume of traffic, registering several million unique visitors per month. People posting on the forums were not identifiable and all posts were readily visible to members of the public. The forums were not moderated and there were no known professional contributors. The extracts presented in this paper are presented in an unedited format.

The forums relating to LS were found by typing “lichen sclerosus” into the websites integrated search function. The threads with the highest volume of discussion were identified and the entire content of the four most popular threads were reviewed. The threads were active between 2011 and 2017. In total 202 posts were reviewed. The posts were copied into a separate document for analysis. Each forum member was assigned a label e.g. FM1 to enable identification of their contribution.

Thematic analysis was the chosen method for the qualitative investigation due to its accessibility and flexibility. This flexibility allows this method to be applied to a range of data to generate a meaningful analysis [11]. The data were analysed following the Braun and Clarke [11] framework for thematic analysis. A researcher (GB) read and re-read the posts to become familiar with the dataset. Basic codes were assigned manually using an inductive approach. Other researchers (SH, LB) reviewed large sections of the dataset and codes; no amendments were made to the coding frame. GB then completed the coding and codes were collated into themes using a mind-mapping tool [12]. All data relating to each theme were then reviewed and collated into distinct documents. Themes were then revised into broader descriptive and conceptual clusters using thematic maps. These were reviewed and confirmed by all researchers. GB is a Gynaecology Registrar caring for women with Vulval Lichen Sclerosus. Consequently, her position and perspective may influence the research. In acknowledgement, a reflexivity diary was completed by GB at each stage of the analysis. LB, however, has no previous background in this area and her involvement in the coding process afforded a degree of analyst triangulation.

This analysis is a reflection of the experiences and reality of individual participants as in a realist approach. The analysis also offers an insight into how these experiences and realities are an effect of society, for example, the impact of social discourse upon how women experience their bodies.

### Ethical considerations

The data were posted on an open forum that did not require permission to access. The posts were visible to members of the public. As the forums contained publicly available information, consent was not necessary [13]. As per BPS ethical guidance [10], to maintain confidentiality and anonymity we have not included Internet pseudonyms. It would be possible for readers to identify the Internet identity of some the authors of the quotes by searching for the direct quotes online. Therefore, to maintain anonymity, a small number of quotes have been slightly amended to prevent this from happening. If quotes were altered this was done in a way that maintained the original sentiment of the comments. General principles on the ethics of internet-based research are discussed in detail elsewhere [10, 13, 14].

## Results

In total, 202 posts spanning a six-year period were analysed. There were 73 people who posted on the forums. Five key themes emerged, exploring problems with obtaining a diagnosis, rationalisation and validation of women’s experience, self-management of the condition, seeking advice and guidance, and the social repercussions of the disease. Table 1 demonstrates the forum members with quotes illustrating each theme.

**Table 1:** The forum members with quotes illustrative of each theme

Theme	Quotes illustrating theme
Theme 1: Problems obtaining a diagnosis	FM1, FM2, FM6, FM8, FM12, FM13, FM18, FM19, FM21, FM23, FM26, FM27, FM29, FM30, FM31, FM43, FM50, FM52, FM 59, FM65, FM68
Theme 2: Rationalisation and validation of their experience	FM2, FM6, FM8, FM12, FM19, Fm21, FM24, FM25, FM32, FM38, FM43, FM48, FM50, FM52, FM54, FM56, FM60, FM65, FM68, FM73
Theme 3: Seeking control over their condition	FM2, FM5, FM6, FM8, FM10, FM11, FM12, FM16, FM17, FM19, FM20, FM21, FM22, FM25, FM26, FM30, FM37, FM38, FM42, FM43, FM44, FM45, FM47, FM48, FM50, FM51, FM52, FM53, FM54, FM55, FM61, FM69, FM70
Theme 4: Sharing and seeking advice and guidance	FM2, FM5, FM6, FM8, FM9, FM10, FM11, FM12, FM14, FM16, FM17, FM19, FM20, FM21, FM22, FM23, FM25, FM26, FM27, FM29, FM30, FM34, FM35, FM36, FM37, FM38, FM39, FM41, FM42, FM43, FM44, FM45, FM46, FM47, FM48, FM49, FM50, FM51, FM52, FM53, FM55, FM57, FM58, FM59, FM60, FM61, FM62, FM64, FM67, FM68, FM70, FM71, FM73
Theme 5: Social repercussions	FM1, FM2, FM6, FM11, FM12, FM13, FM17, FM18, FM19, FM20, FM24, FM26, FM30, FM34, FM37, FM41, FM43, FM46, FM50, FM51, FM52, FM58, FM61, FM66, FM67, FM73

### Theme 1: Problems obtaining a diagnosis

Many women experienced a delay in diagnosis. Often this was a consequence of misdiagnosis with women repeatedly being given treatment for vaginal thrush.

It took about 3 years of going back and forth to GP who at the time said it was thrush and I was wasting her time, it got quite heated at times because I knew it wasn't thrush so took myself off to hospital who took biopsy and came back as LS. (FM21)

Women attributed delays in diagnosis and misdiagnosis to primary care professionals’ lack of knowledge and experience.

I am happy to have a diagnosis at last but to be honest I left the surgery feeling really p***ed off! For some reason it really annoyed me that the Dr diagnosed me and developed a medical management plan so god damn easily!! I must have seen some shockingly c*** Dr's before as this one has seen and treated LS & HS before, seems to know what to do about it - why oh why couldn’t I have met this Dr all those years ago????? (FM6)

In addition to a lack of knowledge and experience, women also reported that health professionals were dismissive of their symptoms, did not listen to their concerns and had labelled them as “time-wasters”. Women expressed a reluctance to present to Health Professionals.

I was diagnosed in Sept 2010 after months and months of doctors not knowing what the problem was, mistreating it or just dismissing it (FM65)

Other women found it difficult to communicate their symptoms and experienced anxiety and embarrassment related to seeking healthcare advice. This resulted in expressions of reluctance to seek a medical opinion and contributed to a delay in diagnosis.

I cannot believe I have stumbled upon this thread…I have been suffering silently from all of the symptoms mentioned for years, and have been too embarrassed to see my GP. (FM68)

Women expressed a mixture of emotions upon receiving a diagnosis. While some expressed relief at being given an explanation for their symptoms, others expressed frustration over the time they had spent suffering without explanation.

I cried when she made the diagnosis, sheer relief to be honest. All that time I felt like I was over-reacting. I have steroid cream but need to use it rarely now. That gynaecologist changed my life, I will always be thankful, and I recommend seeing one if a doctor isn't any help. (FM65)

### Theme 2: Rationalisation and validation of their experience

Women sought to understand why they had developed the disease. Having an understanding of what causes LS allowed women on the forum to align this with their health beliefs, make sense of their experiences, and sometimes to receive vindication from a sense of blame for their symptoms.

I am really into looking at the cause for it, although I know that they cannot be really specific of the cause. Some say its linked with an autoimmune disorder, hormonal, etc. etc., I do know that for many months I have been feeling so exhausted?? (FM2)

I was diagnosed with LS a few months ago and I have to admit I felt dirty at first. I don't sleep around and had only about five sexual partners so it came as a bit of a shock. Reading more about it it doesn't seem to be anything to do with my lifestyle but just one of those things. (FM25)

Some women expressed reassurance in knowing why they had been getting symptoms. They now had an explanation to validate and explain their experiences.

I know this is a slight resurrection of a thread but I'm recently diagnosed too. I've avoided sex for years as it was uncomfortable, now I know why!!! (FM52)

I am 38 (to the person who asked) and I remember itching myself until I bled when I was in the pubertal stages. It all makes sense now. (FM43)

Other women shared their symptoms with other forums members to corroborate and compare their experiences with those of their fellow sufferers.

I have got a couple of sores that are really, really itchy, especially at night, and I scratch so much they bleed. Although at first the scratching is kind of nice as it's such a relief, if that makes sense?!? (FM68)

How does all your LS manifest? Mine was an itchy, hot sore fanny with lots of little white pimples (which I thought were milia) and thickened areas, this is the first symptomatic flare and I do appear to have lost some of my labia minora (FM52)

### Theme 3: Seeking control over their condition

One of the most common threads of discussion focused on attempts to identify triggers for an episode of LS. Women on the forum wanted to avoid suspected causes to reduce their flares and share self-management strategies to gain control over their condition.

I put on Replens three times weekly using an applicator and if I have sex I use 'Yes' lubrication. This holiday I've purchased shorts a size bigger to be more comfortable and give me more freedom and the thong underpants were thrown out months ago. Big Pants! Don't despair and make little changes. I've transitioned from being devastated to accepting that, as awful and life changing as Lichen Sclerosis is, it doesn't define me. (FM46)

Knowledge is a key asset that allows women to understand their condition and gain some control. Women used various tools to research their condition, including using the forums.

After years of problems due to Lichen Sclerosus, i.e. itching, splitting skin, adhesions and fusing, I had had enough. So, just over a year ago after doing some research on the internet, I found an article on http://www.lichensclerosus.net I went to my GP with the article (FM 49)

Over half the discussions on the forum related to self-management of LS. There was evidence of women self-managing their condition through discussions with their medical team, such as requests for medication, seeking referral and planning follow-up schedules. This entailed taking an active and assertive patient role in their relationship with General Practitioners.

The link I popped on higher up, suggests a hormone cream, this is supposed to heal damaged tissue. So I might nip to my GP and discuss with her about it. (FM2)

I am going to ask my GP about regular reviews though as I am concerned that was never offered to me. (FM6)

I am waiting to speak to GP so I can be referred to a vulval clinic, as I don't want my GP to manage it - as they have no idea really. (FM12)

### Theme 4: Sharing and seeking advice and guidance

Forum members valued each other’s opinion and knowledge. Women utilised the lived experience of fellow contributors to answer the questions healthcare professionals did not have the answers to. Some women expressed a lack of confidence in their healthcare professionals and sought advice from the fellow sufferers instead.

Please tell me what you mean by "clear up"? Does it go away or is the skin ruined never to recover? Doctors say "maybe" and "don't know". (FM71)

I have an appointment to have a biopsy in May, we are meant to be going on holiday with friends a week later, has any one had this biopsy done? Is it painful, would I be in pain after the biopsy, I am so worried, my husband said I should cancel the holiday straight away, get this sorted first, I would really like to hear from anyone who can advise me (FM35)

A large proportion of forum members sought advice on possible therapies and women were keen to share the results of different regimes they had tried. This was related to both alternative therapies and prescription medications. Many women had tried multiple different therapies.

I try allsorts, aqueous cream can keep area soothed when not a big flare up.

Sanitary products make it worse, but are necessary. Sex can be painful, but lube is helpful. I reckon it has a link with gluten, sugar etc. for me. I cannot eat a lot of bread products and when I went on a low gluten diet my symptoms were better.

Am going to try Evening Primrose Oil supplements and Omega 3 (cannot remember site I saw this advice) and to cut down on sugar and gluten in my diet. (FM10)

There was evidence that women utilised the advice given by others.

Your remedies are amazing! Could teach my doc a thing or two! I just tried whatever random thing the GP prescribed, only the steroid cream has helped. (FM6)

### Theme 5: Social repercussions

Lichen Sclerosus predominantly affects women’s vulva. Discussing issues related to genitalia with family or friends is difficult for some women on the forum due to socio-cultural barriers. Women reported having nobody to turn to for advice or support and find it hard to raise the subject. The forum was valued as a place to express their concerns.

Im sorry to hear you have had to suffer in silence with this. It can be a very sensitive and embarrasing subject to talk about (FM2)

It's nice to have others to chat to about it, it's hardly a subject to bring up at the school gates or among friends, and I don't have any close female relatives I could talk to about it. (FM65)

A predominant symptom of LS is severe pruritis. Women on the forums frequently experience a desire to scratch. Women report that this can be embarrassing and socially inconvenient.

It's been so uncomfortable and hopefully after reading this post I can try other remedies too - I suffer with constant itching, it's awful.. and at times embarrassing! (FM30).

Another consequence of LS is that it can lead to painful sexual intercourse. Women reported that this impacts upon their sexual interactions and subsequently can lead to relationship breakdown.

It has been difficult trying to explain to my husband "it’s not that I don't fancy you or I have gone off sex, its just that it hurts too much" I have since got him to read loads of LS forums and now he understands! Basically, get your hubby to read other peoples comments about LS, it might just help. (FM18)

When I was diagnosed I felt very alone and upset.( I do not now have a partner as one reason he left was due, he said, to us having ceased to have sex as the tearing etc. was painful) (FM20).

LS is relatively unheard of and most people will not know someone with the same diagnosis. Additionally, the intimate nature of the disease may prevent women from discussing their experiences. Some women reported that the forums were the only place they were able to discuss their concerns with other women with LS.

I am so relieved to find this site and read all your posts to realise I am not alone in this, I have suffered with LS for 3 years now and it is really affecting me emotionally. (FM24)

### Language used to reference genitalia

Although not a theme in itself, it is worth noting the colloquial language used by the women to reference the female genitalia. Few forum members use the term vagina, vulva, labia or genitalia despite the anonymity conveyed by the forum.

I still love you, even though you've got leprosy of the tuppence (FM4).

I stand tall in my fight for a pleasant looking lady garden (FM2)

I've just read through the thread and no one else has mentioned this, but (TMI) some of my lady bits have almost disappeared (FM69)

Did anyone see Embarrasing Bodies last night? They were discussing Foof problems & mentioned LS. (FM6)

It's definitely LS, my fanny looks like it's seen a ghost (FM52)

## Discussion

This study gives insight into the dialogue between women with vulval LS. These conversations deal with managing the condition, practical day-to-day issues and the emotional and social impact of living with LS. The women in our analysis experience delays obtaining a diagnosis. This vital step permits women to validate and rationalise their experience of living with their bodies. Women with LS utilise a variety of media to actively self-educate on their condition, in particular, they value the life-experience of women on the forum to advise and inform. Women use this knowledge to manage their condition and play an active role in partnership with HCPs. The women on the forums report negative social repercussions as a consequence of LS. Online forums offer freedom and community enabling women to find connections and openly discuss their intimate problems.

An Eve Appeal survey, “Take the Vulva Vow”, revealed that only 1% of parents use the word vulva when talking to their daughters about their genitalia [15]. This is thought to be due to the associated stigma attached to the terms vulva, vagina or labia. Additionally, people demonstrate poor knowledge of the female anatomy; a recent Yougov poll revealed that 59% of men and 45% of women were unable to identify the vagina on a diagram of the female genitalia [16]. This is compounded by texts such as The Body Book [17], which refers to the “baby-making hole”. The colloquial language used by the forum members reflects a cultural tendency to avoid using correct anatomical terms. The taboo of discussing women’s health [18] and confusion over female anatomy are barriers to seeking advice from HCPs, family and friends. Our analysis demonstrates that women not only find it difficult to talk about their genitalia, they also are embarrassed by HCPs examining their genitalia. According to a survey conducted by the RCOG, 24% of women reported embarrassment of their body image as a reason not to seek healthcare advice and 21% of women who did not attend for their cervical smear test referenced reasons of embarrassment [19]. The RCOG document “Better for Women” recommends ways to tackle these barriers to healthcare and reduce the taboo attached to women's health, including improvements in the education of young people through the provision of accurate information about women’s health and training teachers to confidently use correct anatomical terms [19].

Women with LS frequently experience difficulties obtaining a diagnosis; in one cohort the mean delay was 5.3 years [5]. For the women in our study, the barriers to obtaining a diagnosis were multi-factorial and complex. Women felt GPs did not address their concerns and lacked the specialist knowledge and experience required to diagnose LS. Repeated misdiagnoses, in particular vulvo-vaginal candidiasis, resulted in women expressing reluctance to seek advice from primary care physicians. In addition, the stigma attached to discussing women’s health and the embarrassment of being examined by HCPs may contribute to a delay in diagnosis. A prompt diagnosis is important to minimise the biopsychosocial impact of the condition [4]. Additionally prompt diagnosis is vital to limit the physical symptoms, reduce scarring and reduce the risk of developing cancer [20]. There is little evidence looking at the psychological impact of diagnosis upon women with LS. Our investigation demonstrates that receiving a diagnosis can results in a positive impact for women with LS. This correlates with other conditions such as Endometriosis [21]. For women with vulval LS, diagnosis can provide an explanation for their experience leading to relief and validation. It allows women to understand why they were affected and align their health beliefs, such as looking for familial links or re-examining their contributory behaviour. This may allay concerns that lifestyle choices are causative factors. Furthermore, a label for their condition enables women to seek commonality and community other women living with LS.

Our analysis demonstrates that women value knowledgeable and experienced healthcare professionals and want reassurance they are receiving optimal support for their condition. Repeated misdiagnoses and on-going symptoms leads to reduced confidence in primary care physicians. This prompts women to seek referral to specialist services. It is recommended by the British Association of Dermatologists that women with LS should be under the care of professionals with appropriate experience in LS [2]. The RCOG has petitioned NHS England to address the gaps in knowledge and provision of women's health care through the introduction of women's health clinics in community hubs and training on women’s health to primary care [19].

Our data identifies that women with LS value the opportunity to take an active role in healthcare decisions. A model of patient-centred care in which patients play an active role in healthcare decisions has been shown to improve health outcomes [22] and a MORI survey for the Department of Health found that 87% of those with long-term health conditions are interested in playing a greater role in taking care of their condition [23]. HCPs should create an environment that enables women to feel comfortable discussing sensitive issues and facilitate constructive discussion. Identifying women’s priorities for care and nurturing collaborative partnerships with patients will enable HCPs to deliver patient-centred, individualised care. Additionally, women demonstrated attempts to control and manage their condition independently. Research investigating self-management initiatives targeting chronic disease demonstrates improvements in people’s knowledge of their disease and their experience of living with the condition [24]. Online forums are saturated with evidence of women seeking to exert some control over their condition. Evidently, women with LS are motivated to manage their condition. HCPs should build upon his motivation to develop care plans in which the patient is empowered to self-manage their condition.

### Future research directions

Our study identified several knowledge gaps impacting negatively upon women with LS. In particular, women are concerned about the possible triggers that cause a flare of their condition. This is a poorly understood subject with scarce research investigating precipitants for an episode of LS. Several publications demonstrate that maintenance steroids can significantly reduce relapses in LS [20]. However, women are particularly focused on environmental modifications that may reduce flares.

Women are observed investigating medications other that those prescribed by their doctor. They actively seek advice from the forums about different therapies, both conventional and alternative. Treatment for LS is limited to high-potency steroids or disease modulators with no curative treatments identified. As recognised by the LSPSP, women want to know if there are other therapies to treat or even cure their disease [7].

Of the limited research investigating the impact of LS upon women, it is evident that LS can result in significant deterioration in quality of life and sexual function [6, 25]. Women on the forums describe how this impacts their relationships and mental health. For women with long-standing problems with sexual intercourse, treatment of the physical symptoms of LS may not result in a return to satisfactory sexual experiences [26].

Further research is needed to investigate strategies to alleviate the impact of LS upon women’s QoL and sexual function.

Evidence from other chronic conditions demonstrates that online health forums convey benefits to users for example, by improving self-management and self-efficacy [27]. Our study suggests that online forums are highly valued by women with LS. Many women on the forum expressed how valuable it was to discuss their experiences with those suffering with the same condition. However, it is it not known if forums result in improved outcomes for women with LS. Research investigating the impact of online health forums for women with Vulval LS would identify their value in healthcare delivery. In addition, it would be important to identify if outcomes would be improved by implementing moderated forums.

### Strengths and limitations

The study identifies the patient perspective without influencing the discussion. This allowed us to identify what women choose to discuss with their peers. This has resulted in detailed insight into women’s perspective of living with the condition.

Data for the study was collected from a forum of women self-reporting a diagnosis of LS as the nature of the study prohibited us from confirming this diagnosis. Furthermore, we were not able to collect more data in response to emerging themes or directly question participants on their comments. The anonymity of the forum results in researchers not knowing the demographic information of the forum members. Women on the forum self-reported a diagnosis of LS, this could not be confirmed. Consequently, the findings of this study may not reflect the priorities of all women with LS. Not all women with LS will utilise online forums. Other women may use closed forums. Closed forums were not used in this study due to the ethical difficulties of observing forum data as it is not deemed to be in the public domain. Therefore, the conclusions and discussions may not be generalizable to all women with LS. Additional research of more diverse sample is needed to explore this subject further. In addition, Google search results are influenced by previous search history and location. Consequently, there may be other open forums where women discuss Lichen Sclerosus. However, the authors were unable to easily find other open forums in the UK with larger numbers of posts.

## Conclusion

Socio-cultural perceptions make it difficult for women with vulval LS to talk to their family, friends and healthcare providers about their condition. Online forums provide a space for women to connect with each other and engage in conversations about living with LS. These discussions reflect a desire to understand the condition and influence it’s impact upon their lives. HCPs should build upon this motivation and deliver patient-centred healthcare to enable women to be active in the decision-making process and self-manage their condition.

Receiving a diagnosis has positive implications for women with LS. A diagnosis enables women to make sense of their experience and progress their narrative. It is frequent for women with LS to experience a delay in diagnosis. The reasons for this delay are complex and multifactorial. Barriers to healthcare are a consequence of social constraints, communication problems between patient and HCPs and limitations in the knowledge of HCPs. Improving women’s health education for children and healthcare professionals will contribute towards remedying this.

Future research directions that match the priorities of women with LS are essential. In particular, searching for an underlying cause for LS, identifying what triggers an episode of LS and seeking new therapies to control and cure the disease. Women perceive benefit from engaging in online forums. It would be valuable to investigate if this perception translates into measurable improvements in women’s health.

Further information about Lichen Sclerosus can be found at the following sources:

British Association of Dermatologists guidelines for the management of lichen sclerosus, 2018 (http://www.bad.org.uk/shared/get-file.ashx?id=6039&itemtype=document).

British Association of Dermatologists Lichen sclerosus in females—patient information leaflet (http://www.bad.org.uk/shared/get-file.ashx?id=291&itemtype=document).

National Lichen Sclerosus Support Group (NLSSG) (http://www.lichensclerosus.org).

## Data Availability

The datasets analysed during this study are available from the corresponding author on reasonable request.

## Abbreviations

LS: Lichen sclerosus
HCP: Healthcare professional
QoL: Quality of life
RCOG: Royal College of Obstetricians and Gynaecologists
LSPSP: Lichen Sclerosus Priority Setting Partnership
